# The digital signature of emergent tremor in Parkinson’s disease

**DOI:** 10.21203/rs.3.rs-3467667/v1

**Published:** 2023-10-27

**Authors:** Helen Bronte-Stewart, Aryaman Gala, Kevin Wilkins, Matthew Pettruci, Yasmine Kehnemouyi, Anca Velisar, Megan Trager

**Affiliations:** Stanford University; Stanford University School of Medicine; Stanford University School of Medicine; Stanford University; Stanford University; The Smithh-Kettlewell Eye Research Institute; Columbia University College of Physicians and Surgeons

## Abstract

**Background:**

Emergent tremor in Parkinson’s disease (PD) can occur during sustained postures or movement that is different from action tremor. Tremor can contaminate the clinical rating of bradykinesia during finger tapping. Currently, there is no reliable way of isolating emergent tremor and measuring the cardinal motor symptoms based on voluntary movements only.

**Objective:**

Investigate whether emergent tremor during repetitive alternating finger tapping (RAFT) on a quantitative digitography (QDG) device can be reliably identified and distinguished from voluntary tapping.

**Methods:**

Ninety-six individuals with PD and forty-two healthy controls performed a thirty-second QDG-RAFT task and the Movement Disorders Society – Unified Parkinson’s Disease Rating Scale Part III (MDS-UPDRS III). Visual identification of tremor during QDG-RAFT was labelled by an experienced movement disorders specialist. Two methods of identifying tremor were investigated: 1) physiologically-informed temporal thresholds 2) XGBoost model using temporal and amplitude features of tapping.

**Results:**

The XGBoost model showed high accuracy for identifying tremor (area under the precision-recall curve of 0.981) and outperformed temporal-based thresholds. Percent time duration of classifier-identified tremor showed significant correlations with MDS-UPDRS III tremor subscores (r = 0.50, P < 0.0001). There was a significant change in QDG metrics for bradykinesia, rigidity and arrhythmicity after tremor strikes were excluded (p < 0.01).

**Conclusions:**

Emergent tremor during QDG-RAFT has a unique digital signature and the duration of tremor correlated with the MDS-UPDRS III tremor items. When involuntary tremor strikes were excluded, the QDG metrics of bradykinesia and rigidity were significantly worse, demonstrating the importance of distinguishing tremor from voluntary movement when rating bradykinesia.

## Introduction

The cardinal motor symptoms of Parkinson’s Disease (PD) are bradykinesia, rigidity, tremor, gait impairment, and postural instability. Rest tremor is present in over 70% of people with PD (PWP)[1] and occurs at frequencies between 4–7 Hz. Rest tremor is usually suppressed with the onset of action but can emerge during sustained movement (such as walking) and when a posture of the hand or limb is sustained [2–7].

The emergence of tremor during posture holding or voluntary movement can contaminate the clinical rating of bradykinesia using the Movement Disorders Society – Unified Parkinson’s Disease Rating Scale Part III (MDS-UPDRS III), for example during the finger tapping task. In this scale there is no way to isolate involuntary emergent tremor from voluntary movements.

A potential solution to this dilemma is to utilize high resolution sensors to quantify motor behavior. One example of this approach is Quantitative Digitography (QDG), which measures repetitive alternating finger tapping (RAFT) on adjacent tensioned levers, where the hand is held in a sustained posture. QDG provides objective and validated measures of the cardinal motor signs of PD, including rigidity, from a simple 30-second RAFT task performed on a digitography device [8–12]. QDG metrics for bradykinesia, rigidity, and gait impairment are significantly different in PWP compared to age-matched, healthy controls, are correlated with the UPDRS III, the MDS-UPDRS III and respective upper and lower extremity sub-scores, and track the effect of medication, pallidotomy, and deep brain stimulation [8, 9, 12]. Additionally, QDG is effective in tracking symptom progression over time [9, 10, 12, 13]. Critically, initial observations from QDG during periods of tremor suggested that it could capture the unique temporal characteristics of tremor [8]. Therefore, QDG may offer a solution by accurately identifying emergent tremor that could occur in PWP whose hands are held in posture as they perform RAFT, and measure bradykinesia and rigidity more reliably based on voluntary tapping only.

In this study, we demonstrate that emergent tremor has a unique digital signature during QDG-RAFT, which could be reliably identified and could distinguish involuntary from voluntary strikes, enabling more representative measurement of voluntary motor signs such as bradykinesia and rigidity based on voluntary strikes only. We used labelled datasets that were created based on visual identification of tremor during QDG-RAFT by a blinded movement disorders specialist to develop and investigate the efficacy of two methods for identifying tremor on a strike-by-strike basis: 1) Using physiologically-informed thresholds for temporal features of tapping 2) Supervised-learning based XGBoost classifier that uses both temporal and amplitude features of tapping as input features. We performed correlations of tremor percentage detected during RAFT and clinical ratings of tremor severity and persistence from the MDS-UPDRS III. Additionally, we evaluated whether metrics of bradykinesia, rigidity, and arrhythmicity were significantly different when measured based on voluntary strikes only, after the exclusion of tremor. Lastly, we assessed whether the change in metrics was associated with the percent duration of tremor in a trial.

## Methods

### Human Subjects

Ninety-six individuals (52 males) with clinically established PD and forty-two healthy controls (20 males) performed thirty seconds of self-paced, repetitive alternating finger tapping (RAFT) on a digitography device in the off-therapy state. Long-acting medications were withdrawn over 24 to 48 hours and short-acting medications were withdrawn for at least 12 hours prior to testing. For patients with DBS, stimulation was turned off for at least 15 minutes before testing [10]. Data was collected in the Stanford Human Motor Control and Neuromodulation Laboratory. All participants provided written informed consent to participate in the study, which was approved by the Stanford University Institutional Review Board.

### Experimental Protocol

The RAFT involved pressing and releasing tensioned, engineered levers on a digitography device with the index and middle finger in an alternating pattern for thirty seconds, as described previously [9, 10, 12, 13, 14]. Participants were instructed to press and release the keys completely, while going as fast and as consistently as possible for the duration of the trial. The task was performed without auditory or visual feedback: participants closed their eyes and white noise was played through headphones. They were instructed to start and stop tapping only when they heard an auditory cue. The task was performed on each hand. No external cueing was given during the task. Participants also performed the Unified Parkinson’s Disease Rating Scale (UPDRS III). Thirteen individuals performed the original UPDRS III and eighty-three individuals performed the Movement Disorder Society (MDS)-UPDRS III.

### Kinematic data acquisition and analysis

**Figure 1:**
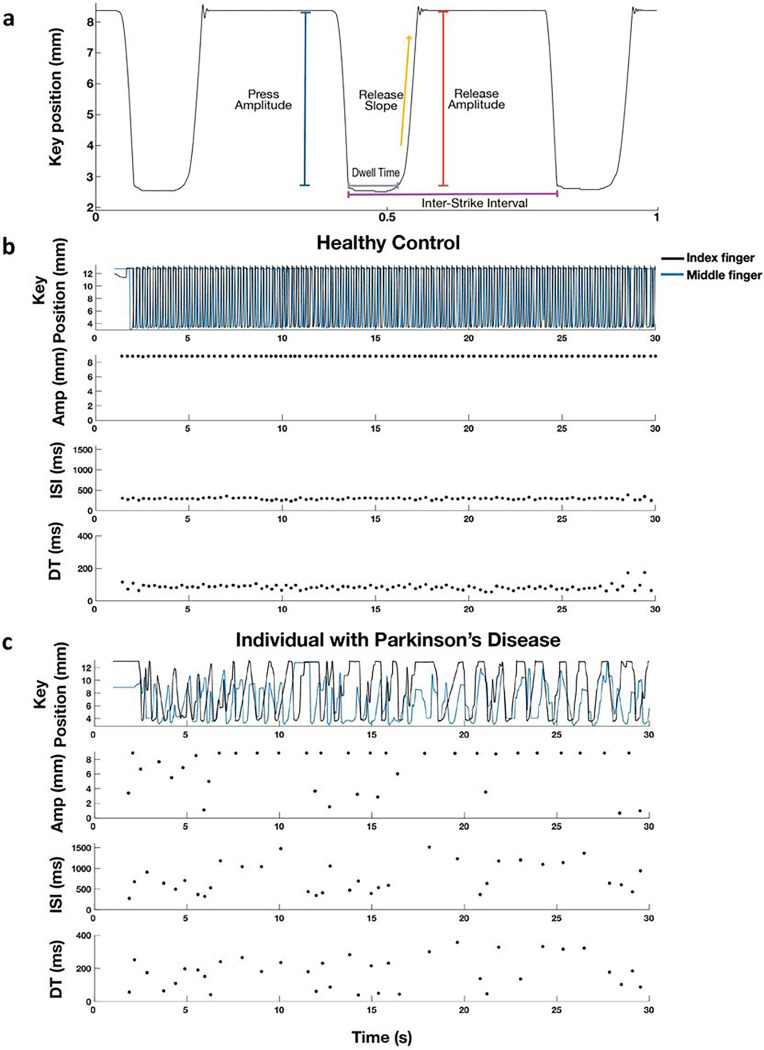
(A) Raw QDG Trace with highlighted features of tapping. Example traces of QDG-RAFT by a (B) Healthy Control and (C) an individual with Parkinson’s Disease. In panels B and C, the first row depicts the key displacement by the index and middle fingers over the 30 second trial, while the second row displays press amplitude and the last two rows depict the inter-strike interval (ISI) and dwell time (DT) of each press by the middle finger respectively.

The digitography device used an optical encoder that produced a voltage signal as the lever was pressed and released, which was linearly related to the displacement of the lever and had a resolution of 62.5um per 40mV [10]. Eight metrics were calculated from the amplitude and timing of the press and release phases of RAFT, as shown in Fig. 1A: press and release amplitudes, inter-strike interval (ISI), dwell time (DT), press and release durations, peak duration, and release slope.

### Visual identification of tremor

An experienced movement disorders specialist (HBS) was shown video recordings of 36 RAFT trials from thirty-one tremor dominant (TD) participants and five akinetic rigid (AR) participants. The rater was blinded to the phenotype of the participant and trials were randomly presented. The rater identified any occurrence of tremor in the trial and provided timestamps for periods of sustained tremor. A labelled dataset was created based on the movement disorders specialist’s ratings, with the positive class (tremor = 1) comprising strikes from five trials in which the rater identified tremor for the entire duration of the trial, and the negative class (tremor = 0) consisting of strikes from 75 trials performed by control participants (Dataset 1).

### Algorithm to identify tremor strikes using temporal characteristics and based on video identification

**Figure 2:**
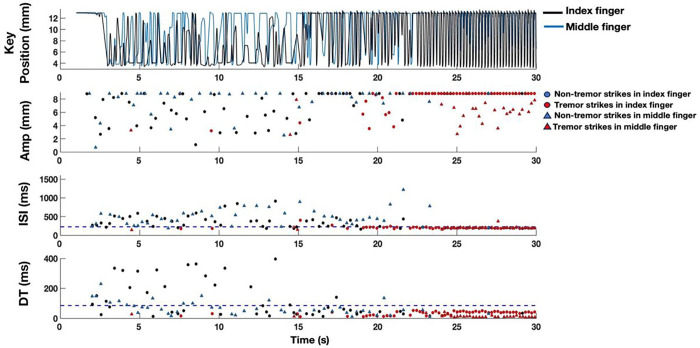
Example of emergent tremor in a Tremor Dominant PD Phenotype. Dashed blue lines represent the ISI (225 ms) and dwell time (85 ms) thresholds investigated to classify tremor strikes.

Visual observation between trials with tremor and trials with no tremor suggested that tremor strikes could be distinguished from voluntary tapping by temporal characteristics of a shorter ISI (higher frequency) and shorter dwell time, Fig. 2, which has been previously observed [8]. Four combinations of physiologically informed ISI and dwell time thresholds were systematically applied to classify tremor in strikes from Dataset 1. The efficacy of each ISI and dwell time threshold combination was evaluated based on the sensitivity (True Positive/True Positive + False Negative) and false positive rate (False Positive/False Positive + True Negative). The most suitable ISI and dwell time thresholds were identified by comparing the resulting sensitivity and false positive rates of each combination of thresholds.

### An XGBoost classifier to identify tremor

An XGBoost classifier was developed to identify emergent tremor in the RAFT trial on a strike-by-strike basis. For each strike, eight features of tapping were extracted as potential input features for the model, which included temporal metrics of tapping: ISI, dwell time, release duration, press duration, peak duration, release slope, and amplitude metrics: press and release amplitude. For the development of the model, tremor strikes from three additional trials, for which the rater provided a timestamp for tremor onset and the emergent tremor persisted for the remainder of the trial, were added to the positive class of the labelled dataset to reduce the class imbalance (Dataset 2). The dataset was split into a training (75% of the dataset) and test set (25% of the dataset) with stratification to account for the class imbalance and prevent biased representation of classes in either the training or test set, which could lead to misleading evaluation of model performance. Features were normalized such that the data has zero mean and unit variance to mitigate the effects of features having different scales on the model and facilitate better convergence. Variance Inflation Factor (VIF) analysis was performed to evaluate multicollinearity between input features. Peak duration was removed from the dataset to eliminate multicollinearity and allow for appropriate interpretation of feature importance. Bayesian optimization was implemented to perform hyperparameter tuning and stratified K-fold cross validation was used to evaluate model performance on the training set and further adjust hyperparameters. Early stopping was applied while training to prevent overfitting and improve generalization of the model. A prediction probability threshold of 0.35 was used, which implies that a strike was classified as tremor if the probability of a particular strike being tremor was determined to be greater than 0.35 by the classifier, since it was found to increase sensitivity while not increasing the false positive rate substantially. Model performance was evaluated on the test set using sensitivity, precision (True Positive / True Positive + False Positive), F1 score (harmonic mean of precision and recall) and the false positive rate as measures of performance, instead of the general accuracy score to mitigate the imbalanced class distribution from biasing the interpretation of model performance. Feature importance was evaluated using two methods: permutation importance and gain importance. Permutation feature importance analysis, using the F-1 score as the scoring metric, computes the decrease in the F1 score when the values of each feature are randomly shuffled. Gain importance measures the gain (reduction in the model’s loss function) achieved by splitting on a particular feature during the construction of decision trees, providing quantitative insight into the contribution of each feature to the model’s overall performance.

### Statistical Analysis

Statistical analyses were performed on MATLAB (version R2022a, MathWorks Inc., Natick, MA, USA). A two-sample t-test was used to evaluate the difference in ages between the control participants and PWP. A Kolmogorov-Smirnov test was used to assess the difference between the distribution of the eight metrics of tapping for tremor and voluntary strikes. A significant threshold of p < 0.006 (0.05/8) was established to account for the evaluation of eight metrics based on a Bonferroni correction. Spearman correlations were used to assess correlations between four calculated tremor sub-scores from the UPDRS and the percent duration of tremor in each trial identified by the XGBoost classifier. An overall weighted tremor score was derived by combining the postural and kinetic sub-scores of the MDS-UPDRS III assessment, along with an equally weighted sum of rest tremor severity and constancy items (i.e., postural tremor + kinetic tremor + 0.5(rest tremor severity + constancy of rest tremor)). A weight of 0.5 was used to give equal weighting to rest tremor severity and constancy scores to prevent rest tremor items from biasing the scores. The significance threshold was set at p < 0.0125 (0.05/4), applying a Bonferroni correction to account for the evaluation of four tremor sub-scores. A Wilcoxon signed-rank test was used to evaluate whether there was a significant change in QDG metrics for bradykinesia (ISI, press amplitude and press amplitude CV), rigidity (release slope), arrhythmicity (ISI CV) and dwell time after the exclusion of tremor in 85 trials performed by TD individuals. Spearman correlations were used to evaluate the correlation between the percent duration of tremor in a trial and the change in metrics after tremor strikes were excluded. The significance threshold for this analysis was set at p < 0.008 (0.05/6) due to a Bonferroni correction to account for the evaluation of the six metrics.

### Code availability

The underlying code for this study [and training/validation datasets] is not publicly available but may be made available to qualified researchers on reasonable request from the corresponding author.

## Results

The mean age of the PD participants was (65.1 ± 9.1 years), disease duration was 8.7 ± 5.7 years and the mean off therapy MDS-UPDRS III (N = 83) was 25.1 ± 13.4. The healthy controls were not significantly different in age (60.0 ± 9.0 years, p = 0.127).

### Characteristics of tremor captured on the digitography device

RAFT performed by a representative, healthy control was characterized by consistent full amplitude and low (short) ISI key strikes with low (short) dwell times as shown in Fig. 1B. On the other hand, RAFT performed by a representative PWP, shown in Fig. 1C, demonstrated fewer taps per thirty seconds, lower amplitudes, greater (longer) ISIs, longer dwell times and more variability in all metrics than those demonstrated by the healthy control.

Figure 2 (see Supplementary Video 1) demonstrates the emergence of tremor in a PWP during RAFT that eventually replaces any voluntary tapping. The RAFT trace initially demonstrates voluntary alternating strikes, characterized by inconsistent amplitudes, irregular, long ISIs and variable, long duration dwell times.

More consistent, non-alternating, high frequency, short dwell time and high amplitude strikes appeared after twenty-two seconds; the synchronized video demonstrated regular, rhythmic tremor of both fingers, which were striking the device at the same time. Most of these strikes had ISIs of ~ 225 ms and dwell times of ~ 55 ms.

### Using temporal characteristics to distinguish tremor from voluntary strikes

The characteristic frequency of PD resting tremor is 4–7 Hz, which is equivalent to an ISI between 143–250 ms. Four combinations of physiologically informed ISI and dwell time thresholds were iteratively applied to classify tremor in Dataset 1. The sensitivity rate and false positive rates were evaluated to determine the efficacy of each combination of ISI and dwell time thresholds. An ISI threshold of 216 ms and dwell time threshold of 70 ms had the lowest sensitivity rate of 50.00% and a false positive rate of 4.30%. Increasing the ISI threshold to 225ms and the dwell time threshold to 85 ms increased the sensitivity rate to 73.45% and false positive rate to 7.49%. We found that further increasing the ISI threshold from 225 ms to 230 ms, while keeping the dwell time constant at 85 ms, increased the sensitivity rate by 2.26% and the false positive rate by 1.23%, whereas increasing the dwell time threshold from 80 ms to 85 ms, while keeping the ISI threshold constant at 225 ms, increased the sensitivity by 5.26% and the false positive rate by 0.67%. Since increasing the ISI above 225 ms lead to an increase in false positive rate without a substantial increase in the sensitivity rate, an ISI threshold of 225 ms and dwell time threshold of 85 ms was chosen for further evaluation (Table 1).

Applying the determined ISI threshold of 225 ms and dwell time threshold of 85 ms to classify strikes in the test set (25% of Dataset 2) had a sensitivity rate of 79%, precision rate of 63%, but a high false positive rate of 6.7%. The high false positive rate was largely due to the ability of some healthy controls to perform voluntary RAFT at a high frequency with a short dwell time.

### Leveraging temporal and amplitude features of tapping increased the accuracy of tremor identification

**Figure 3:**
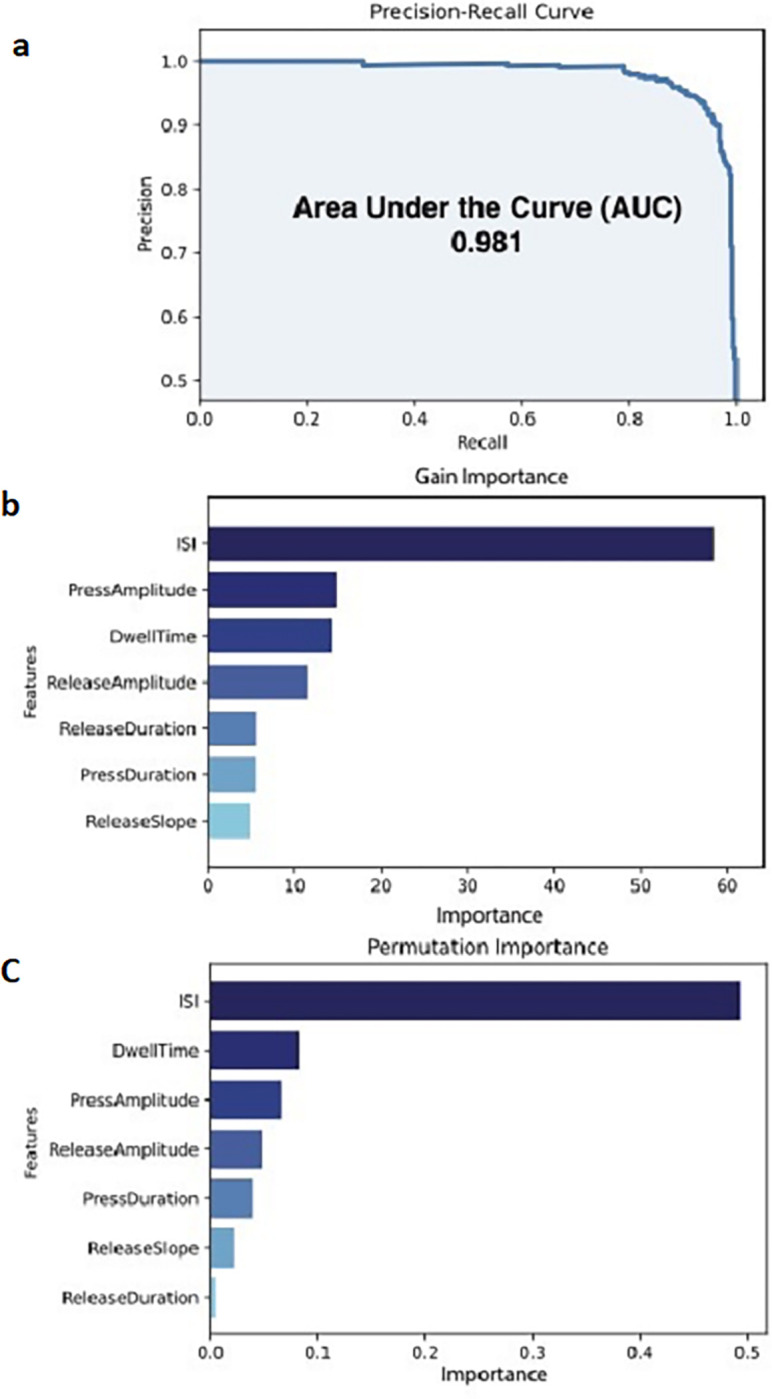
Area under the precision-recall curve depicting XGBoost model performance. B) Evaluation of feature importance based on increase in gain (reduction in loss function) when a particular feature is used in decision trees and C) permutation importance

An XGBoost classifier model was used to identify tremor on a strike-by-strike basis, which included the press and release amplitude as features, in addition to five temporal metrics of tapping. Assessing the XGBoost model on the test set revealed that the classifier had a high sensitivity of 98%, a precision rate of 84%, an area under the precision-recall curve (AUC) of 0.981, and a lower false positive rate of 2.7% (Fig. 3A).

The classifier was further validated by comparing the model’s identification of tremor in a trial to the ground truth labels provided by the blinded movement disorders specialist. The classifier correctly identified the presence of tremor in 31 out of 31 trials visually rated as having tremor by the movement disorders specialist and did not identify any instances of tremor in 4 out 5 trials marked as having no tremor. Only one trial visually rated to have no tremor was identified to have tremor for 0.37% of the trial by the classifier.

### Temporal and amplitude metrics are important features to the XGBoost classifier’s performance

Figure 3B demonstrates that the ISI had the highest contribution to the gain of the model when used in decision trees (58.39). The press amplitude had the second-greatest contribution to the gain of the model (14.84) which was closely followed by the dwell time (14.28) and release amplitude (11.46). The release duration (5.52), press duration (5.48), and release slope (4.76) exhibited a comparatively lesser impact on the model’s gain when included in decision trees.

Evaluating the importance of features based on permutation importance revealed that the four features of tapping that had the highest impact on the F1 score were the ISI (49.2), dwell time (8.2), press amplitude (6.6), and release amplitude (5.2). These were followed by the press duration (4.8), release slope (3.8) and release duration (2.2, Fig. 3C).

### Digital signatures of tapping are different between tremor and voluntary strikes

After applying the classifier to identify tremor in the cohort of RAFT trials performed by 96 PWP, we found that key-strikes classified as tremor had a significantly different distribution of all eight metrics when compared to voluntary strikes; the largest difference was for ISI (D = 0.88) followed by dwell time (D = 0.63), peak duration (D = 0.45), release duration (D = 0.44), release slope (D = 0.30), press duration (D = 0.19), press amplitude (D = 0.18), and release amplitude (D = 0.18), all with P < 0.001 (Table 2). This finding demonstrated that tremor during RAFT had a unique signature that could be measured using QDG technology, which included both temporal and amplitude features.

### Classifier-identified tremor correlated with clinical ratings of tremor

The duration of tremor during RAFT (expressed as a percentage of the time of the trial, %T) was compared to tremor related items in the MDS-UPDRS III from the corresponding upper extremity, in cases where the sum rest tremor and postural tremor items was greater than 1 (n = 59).

The strongest correlation was seen between %T and the weighted overall MDS-UPDRS III tremor score (postural + kinetic tremor + 0.5(rest tremor severity + constancy of rest tremor); rho = 0.50, p = 2.34e-4). %T was also significantly correlatedwith the sum of rest tremor severity + constancy scores (rho = 0.45, p = 0.0012),with the constancy score itself (rho = 0.43, p = 0.0019), and with the sum of rest + postural tremor scores (rho = 0.35, p = 0.0068).

### Significant change in QDG metrics after exclusion of tremor strikes

In trials performed by TD individuals (n = 85), measurement of QDG metrics for bradykinesia (ISI (z = 6.27, p = 5.17e-12), press amplitude (z = 3.92, p = 9.00e-05) and press amplitude CV (z = −3.12, p = 0.0018)), rigidity (release slope (z = −3.35, p = 8.15e-04)) and arrhythmicity (ISI CV (z = −6.04, p = 1.56e-09)) based on voluntary strikes only, showed that there was a significant change in the metrics when tremor strikes were excluded. The corrected values for all of the QDG metrics demonstrated that inclusion of tremor strikes would have masked or under estimated the severity of bradykinesia and rigidity (Table 3). Two trials that had less than five voluntary strikes remaining after the exclusion of tremor were not included in these analyses.

#### Change in QDG metrics correlated with severity of tremor in trial

**Figure 4:**
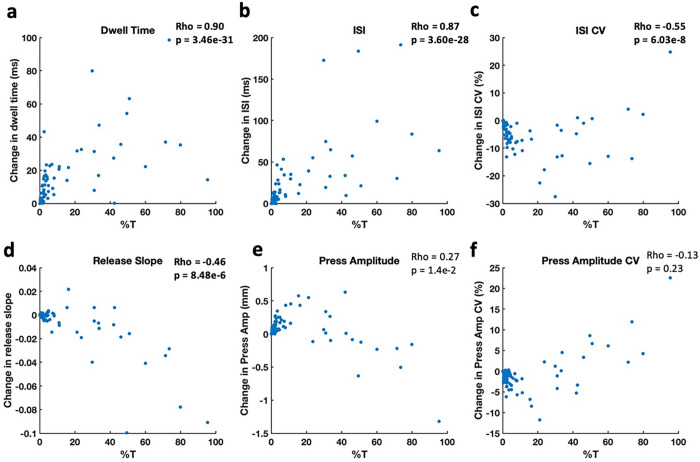
Scatter plot of percent duration of tremor in trail (%T) and change in metrics measuring bradykinesia (ISI, press amplitude and press amplitude CV), rigidity (release slope), arrhythmicity (ISV CV) and dwell time. There was a significant correlation between press amplitude (Rho = −0.69, p = 1.17e-4) and press amplitude CV (rho = −0.63, p = 6.53e-4) with %T when trials with <5% %T were excluded from the analysis (n = 26)

The change in average dwell time (rho = 0.90, p = 3.46e-31), ISI (rho = 0.87, p = 3.6e-28), ISI CV (rho = −0.55, p = 6.03e-08) and release slope (rho = −0.46, p = 8.84e-06) were significantly correlated to % T after the exclusion of tremor, Fig. 4. There was no significant correlation between press amplitude, press amplitude CV and %T.

Out of the 85 TD trials evaluated, 56 trials showed an increase in press amplitude after the exclusion of tremor strikes, which suggested that tremor strikes were lower amplitude compared to voluntary strikes in these trials. We found that majority of these trials (36 out of 52 trials) had < 5% %T in the trial. In contrast, 11 trials had a decrease in press amplitude after the exclusion of tremor strikes, which indicated higher amplitude tremor strikes; these trials had > 20%T, Fig. 4. When the trials with < 5%T were excluded, there was a significant correlation between the %T and the change in press amplitude (rho = −0.69, p = 1.17e-4) and press amp CV (rho = 0.67, p = 2.80e-4); the direction of change showed that including trials with > 5% tremor underestimated the hypometria and variability of the amplitude of voluntary tapping. There was still a significant correlation with release slope (rho = −0.63, p = 6.53e-4).

## Discussion

Emergence of rest tremor of the upper extremity was reliably detected and characterized when the hand was in a sustained posture and the index and middle fingers were in contact with tensioned, engineered levers of a digitography device (Quantitative DigitoGraphy, QDG) in people with Parkinson’s disease (PWP). A model incorporating both temporal and amplitude features of lever displacement accurately differentiated involuntary tremor strikes from voluntary finger tapping strikes during repetitive alternating finger tapping (RAFT). The characteristics of the tremor strikes were significantly different from voluntary strikes in all eight QDG metrics of tapping. The inclusion of amplitude as well as temporal features to distinguish tremor from voluntary strikes resulted in a higher sensitivity (98%) compared to the best model using only temporal features (79%) and lowered the false positive rate from 6.7–2.7%; the area under the precision-recall curve (AUC) was 0.981. The higher false positivity rate seen in the comparisons using only temporal features was due to the performance from a few healthy controls, who could voluntarily tap at high frequencies with short dwell times for the duration of the task. The percent time that tremor was detected in PWP during QDG-RAFT was significantly correlated with the clinical assessment of tremor using the Movement Disorders-Unified Parkinson’s Disease Rating scale (MDS-UPDRS) III tremor sub-scores. The analysis of the QDG-RAFT trace from tremor dominant PWP after the tremor strikes had been excluded showed that there was significant change in QDG metrics for bradykinesia, rigidity, and arrhythmicity when measured based on voluntary tapping only, and that the inclusion of tremor strikes would have contaminated the measurement of the motor signs. There was a significant correlation between the percent time of tremor in the trial and change in the metrics after exclusion of tremor strikes. This suggests that a more accurate and precise measure of the disorders of voluntary motion in PD (bradykinesia, rigidity, freezing behavior and freezing episodes) can be achieved when tremor strikes are isolated and excluded.

QDG technology was originally introduced as part of a suite of objective motor assessments that were developed to supplement the clinical assessment of PWP, who were evaluated before and after deep brain stimulation along the CAPSIT protocol [8, 15, 16, 17]. QDG-RAFT metrics have been validated with the UPDRS III and MDS-UPDRS III scores and sub-scores of bradykinesia (upper and lower extremity), rigidity, gait impairment and Freezing of Gait (FOG) [10–13]. This study has shown that tremor can be reliably measured using QDG technology and correlates with the MDS-UPDRS III tremor sub-scores.

The clinical characterization of tremor is usually divided into rest, postural, or action tremor. However, it is well known that emergent (rest) tremor may occur after the limbs have been held in a position of posture and/or when walking. [2–7]. Action tremor is largely defined as the oscillatory motion of the limb during a voluntary task that is superimposed on a linear or smooth trajectory, such as performing the finger-to-nose-to finger test or when drawing spirals. Emergent rest tremor may occur when a person with PD has their arm held in a posture while they perform a voluntary movement, such as the finger tapping task in the MDS-UPDRS III; the emergent tremor may interrupt or overtake the voluntary tapping. This phenomenon is demonstrated in the video in this study, where an emergent rest tremor of the wrist overtook the voluntary finger tapping on the digitography device (Fig. 2; Supplementary Video 1). The fact that the fingers were resting on tensioned engineered levers that sensed the amplitude and timing of lever displacement allowed the algorithm to distinguish involuntary tremor strikes from voluntary strikes and to determine that there was a different digital signature of tremor strikes. Separating tremor strikes from voluntary strikes provided a measure of the amplitude, frequency of tremor and the constancy of tremor during the thirty second trial that correlated with the MDS-UPDRDS tremor sub-scores.

The emergence of tremor poses the risk of contaminating estimates of bradykinesia, such as during the finger tapping task of the MDS-UPDRS III. Bradykinesia can be under-estimated when tremor takes over voluntary tapping at a frequency that is higher than that seen in voluntary movement; tremor frequency is 4–7 Hz, while voluntary tapping in PWP is usually between 1–3 Hz. If the PWP is asked to extend both finger and thumb when tapping, the execution of the movement is usually slower and more representative of their bradykinesia.

Bradykinesia can be over-estimated when intermittent tremor can cause irregularities in voluntary finger tapping that may be graded as hesitations, which are designed to be reflective of the sequence effect of bradykinesia.

This was confirmed by the results of this study: tremor strikes were found to artificially reduce ISI (i.e., tapping appears faster) when not accounted for. Tremor also was found to affect other aspects of bradykinesia, such as amplitude and rhythmicity. Tremor strikes in trials with intermittent emergent tremor (<%5%T) had a smaller amplitude in relation to the voluntary strikes, thus artificially decreasing the observed amplitude, if not excluded. Whereas, in trials where emergent tremor overtook voluntary tapping for substantial portions of the task, tremor was characterized by high amplitude strikes which artificially increased the observed amplitude, if not excluded. This finding suggested that the severity of bradykinesia would have been overestimated without the removal of tremor strikes in trials with minor intermittent emergent tremor. On the other hand, bradykinesia would have been underestimated in trials with prolonged emergent tremor if tremor was included in the analysis. Currently there is also no way to distinguish emergent tremor that overtakes voluntary tapping on smart phones, which will lead to underestimating bradykinesia from smart phone apps that only count the number of taps per minute [18]. This study demonstrated that inclusion of tremor strikes in analysis of the QDG-RAFT trial would have significantly under-represented the severity of bradykinesia and rigidity.

### Complimentary technologies

A remote continuous passive measure of resting tremor in PD is available using inertial measurement units (IMUs) and/or accelerometers embedded in commercial smartwatches, which are usually worn on the more affected wrist [19–25]. Either tremor acceleration or the mean daily time spent with tremor was correlated with certain aspects of the MDS-UPDRS III tremor sub-scores.

The accelerometer in smartphones has also been used to measure rest and postural tremor frequency by holding the phone in one hand and tremor measured by a smartphone was correlated to that measured by needle EMG [26]. Lipsmeier et al. reported on the first clinical trial in which the smartphone was used in a variety of active tests and passive monitoring over a six-month period [27]. Rest tremor was measured when the participant held the smartphone daily for thirty seconds at a time. The average of 2 weeks of daily testing demonstrated that the skewness of rest tremor moderately correlated with the MDS-UPDRS III rest tremor constancy score. Other features of tremor were not reported. In the clinic setting a tremor stability index measured from a wearable accelerometer differentiated essential tremor from Parkinson’s disease tremor but no comparison to the clinical rating scale of tremor was provided [28].

Passive monitoring relies on distinguishing tremor from voluntary movement by spectral (frequency) analysis and usually long periods of recordings (six days to two years) are used to generate a mean daily tremor occurrence [23, 24, 27, 29]. While useful for measuring tremor over time, passive monitoring studies have also suffered from limitations in compliance of use, especially as the period of monitoring became longer [30–33]. This may be solved by the use of the Apple watch, which also provides many other functions thus perhaps making it more desirable to wear. In addition to traditional spectral analysis, machine learning and deep learning techniques, which are capable of capturing more complex patterns in time and frequency domain features extracted from inertial signals, have been implemented to improve the accuracy of tremor classification in real-world settings [34]. While these approaches have shown promise, discriminating certain voluntary movements from tremor was found to be challenging and makes them susceptible false-positives [23].

The current study demonstrated that QDG technology distinguished tremor from voluntary movement using only a 30 second task, and the percent time with tremor significantly correlated with the MDS-UPDRS tremor sub-scores. The short period of time to perform the RAFT task may improve compliance compared to passive monitoring devices, which is supported by a previous study showing a compliance rate of 91% when participants with early-stage untreated PD were asked to perform RAFT among other tasks once a week for six months [14].

### Limitations

There is a risk of overfitting for the XGBoost model due to the somewhat small dataset in the current study. We used a 75%/25% train/test split to minimize the potential of overfitting. Additional labelled tremor strikes from more participants would reduce potential overfitting by adding variability to the dataset and improving generalizability. The reported sensitivity and false positive rate of the temporal thresholds may be overestimated due to data leakage. Rather than using a train/test split, these thresholds were determined by comparing the sensitivity and false positive rates across various ISI and dwell time thresholds using the same dataset. This only further supports the advantage of the XGBoost model approach for identifying tremor. The observed correlations between MDS-UPDRS tremor sub-scores and the model-identified tremor characteristics may have been slightly deflated due to having multiple certified UPDRS raters in this cohort, thus introducing inter-rater variability in the MDS-UPDRS scores, a known limitation even among certified raters [35]. Despite this potential variability, significant correlations were still observed. Lastly, although it may be argued that passive monitoring over days or weeks may be more likely to capture tremor compared to a 30 second task, we demonstrated that tremor was reliably captured and measured. Indeed, the performance of RAFT was able to elicit tremor even in participants with an MDS-UPDRS III tremor score of 1. This may be due to the known influence of cognitive load on tremor amplitude [36, 37].

## Conclusions

The paper demonstrated that emergent tremor has a distinct digital profile that can be distinguished from voluntary movements when fingers are in contact with tensioned levers. Static temporal thresholds were less effective in differentiating high frequency voluntary tapping by healthy controls from tremor in PWP, leading to a high false positive rate. On the other hand, an XGBoost classifier which uses both temporal and amplitude metrics of tapping as input features reliably identified tremor and discerned voluntary tapping. The percent duration of tremor identified by the classifier in thirty-second QDG-RAFT trials correlated with the MDS-UPDRS III items of tremor. When tremor strikes were excluded from the analysis of the disorder of voluntary tapping there were significant changes in the QDG metrics of bradykinesia, rigidity and arrhythmicity, demonstrating that the inclusion of tremor when assessing voluntary movement will underestimate the severity of Parkinsonism. These findings highlight that tremor can be reliably measured with QDG and completes the evidence that QDG technology produces validated metrics of all the motor signs in PD in real time from a simple thirty second RAFT task on a portable digitography device that can be used remotely.

## Data Availability

The datasets used and/or analyzed during the current study available from the corresponding author on reasonable request.
